# The Spanish version of the Patient-Rated Wrist Evaluation outcome measure: cross-cultural adaptation process, reliability, measurement error and construct validity

**DOI:** 10.1186/s12955-017-0745-2

**Published:** 2017-08-24

**Authors:** Roberto S. Rosales, Rayco García-Gutierrez, Luis Reboso-Morales, Isam Atroshi

**Affiliations:** 1 Unit for Hand and Microsurgery, GECOT La Laguna, Tenerife, Spain; 2 Department of Orthopedics, University Hospital of La Candelaria, University of La Laguna, Tenerife, Spain; 3 Department of Clinical Sciences - Orthopedics, Lund University, Lund, Sweden; 4 Department of Orthopedics Hässleholm-Kristianstad, Hässleholm Hospital, Hässleholm, Sweden

**Keywords:** PRWE, Wrist, Hand, Distal Radius Fracture

## Abstract

**Background:**

The Patient-Rated Wrist Evaluation (PRWE) is a widely used measure of patient-reported disability and pain related to wrist disorders. We performed cross-cultural adaptation of the PRWE into Spanish (Spain) and assessed reliability and construct validity in patients with distal radius fracture.

**Methods:**

Adaptation of the English version to Spanish (Spain) was performed using translation/back translation methodology. The measurement properties of the PRWE-Spanish were assessed in a sample of 40 consecutive patients (31 women), mean age 58 (SD 19) years, with extra-articular distal radius fractures treated with closed reduction and cast. The patients completed the PRWE-Spanish and the standard Spanish versions of the 11-item Disabilities of the Arm, Shoulder and Hand (QuickDASH) and EQ-5D questionnaires at baseline (health status before fracture) and at 8, 9, 12, and 13 weeks after treatment. Internal-consistency reliability was assessed with the Cronbach alpha coefficient and test-retest reliability with the intraclass correlation coefficient (ICC) comparing responses at 8 and 9 weeks and responses at 12 and 13 weeks. Cross-sectional precision was analyzed with the Standard Error of the Measurement (SEM). Longitudinal precision for test-retest reliability coefficient was analyzed with the Standard Error of the Measurement difference (SEMdiff) and the Minimal Detectable Change at 90% (MDC_90_) and 95% (MDC_95_) confidence levels. For assessing construct validity we hypothesized that the PRWE-Spanish (lower score indicates less disability and pain) would have strong positive correlation with the QuickDASH (lower score indicates less disability) and moderate negative correlation with the EQ-5D Index (higher score indicates better health); Spearman correlation coefficient (r) was used.

**Results:**

For the PRWE total score, Cronbach alpha was 0.98 (SEM = 2.67) at baseline and 0.96 (SEM = 4.37) at 8 weeks. For test-retest reliability ICC was 0.94 (8 and 9 weeks) and 0.96 (12 and 13 weeks) with SEMdiff 7.61 and 6.18 and MDC_95_ 13.74 and 12.11, respectively. The PRWE-Spanish scores had strong positive correlation with the QuickDASH scores at baseline (*r* = 0.71) and at 8 weeks (*r* = 0.79) and moderate negative correlation with the EQ-5D Index (*r* = −0.44 and *r* = −0.40, respectively).

**Conclusions:**

The PRWE-Spanish showed high internal-consistency and test-retest reliability and good construct validity in patients with distal radius fracture.

## Background

In evaluating musculoskeletal upper-extremity disorders patient-reported measures of disability and pain are now increasingly used as primary outcomes in randomized trials and observational studies [1]. They are also frequently used in national registries.

The currently available measures that assess outcomes related to the hand and wrist are the Disabilities of the Arm, Shoulder, and Hand (DASH) questionnaire [2], the Michigan Hand Outcomes Questionnaire [3], the Upper Extremity Function Scale [4], the Boston Carpal Tunnel Syndrome (CTS) Questionnaire [5], and the Patient-Rated Wrist Evaluation (PRWE). The PRWE is a joint-specific outcome measure that is widely used in evaluating patients with wrist diseases or injuries [6, 7].

The relative advantage of the PRWE compared to other upper-extremity specific patient-reported outcome measures, such as the DASH, is that the PRWE is wrist-specific and its score is less influenced by possible concomitant shoulder and elbow problems. Besides, it was primarily developed to assess the constructs of pain and disability in patients with distal radius fracture (DRF), the most common facture in the human body [6]. Reviews have concluded that the PRWE is a reliable, valid and responsive measure of pain and disability in patients with DRF and other hand and wrist conditions [8, 9].

Studies that have assessed the reliability of the PRWE have reported intraclass correlation coefficient (ICC) values ranging from 0.78 to 0.94 in patients with different wrist/hand injuries suggesting good reliability [6, 10–13]. While the DASH may be more appropriate for patients with disability and pain in multiple areas in the upper extremity, the PRWE has demonstrated superior validity and responsiveness in patients presenting with pain and disablement only in the wrist or hand [14].

The PRWE has been adapted to many different languages [10–12, 15–25]. Several Spanish-language patient-reported outcome measures related to upper-extremity assessment are currently available, including the DASH, QuickDASH, Boston CTS questionnaire, and CTS-6 [1, 26]. To our knowledge, a Spanish (Spain) version of the PRWE has not been published. The PRWE-Spanish would be a useful patient-reported outcome measure for clinical research in Spain and would facilitate comparison of results from clinical research concerning DRF and other wrist problems.

The aims of this study were: 1) translation and cultural adaptation of PRWE into Spanish (Spain), and 2) preliminary assessment of test-retest reliability, internal-consistency, measurement error, and construct validity in patients with DRF.

## Methods

### Translation and adaptation of the PRWE to Spanish

The translation and adaptation of the PRWE to Spanish followed the protocol, proposed by the International Quality of Life Assessment (IQOLA) project, that has been previously used to obtain the different language versions of the Short Form-36 (SF-36) Health Survey [27, 28] and the Spanish versions of the DASH [1, 29] and the CTS questionnaires [1]. The adaptation process consisted of 2 steps:

1. Forward translation and quantitative evaluation of the difficulty and equivalence of translation by bilingual translators whose original language is the same as that of the target adapted version [1, 27]. The English PRWE was translated into Spanish by four bilingual translators (two of them with clinical experience) whose native language was Spanish. Each translator prepared a separate translation and rated the difficulty of translation on a scale ranging from 0 (no difficulty) to 100 (severe difficulty), and the equivalence of translation from 0 (no equivalence) to 100 (complete equivalence) for each item in these initial Spanish versions [1, 27, 28]. The principal researcher (RSR) and the four translators had a meeting to produce the first Spanish adaptation (version 1.0) after consensus. This version was checked for clarity and comprehension.
2. Back translation, quality control and pretest of the adapted version. The purpose of this step was to ensure concept equivalence between the adapted version and the original. The initial Spanish version (version 1.0) was translated back into English by two bilingual translators (living in Spain) whose native language was American English and both were blinded to the original English version. These two back-translations were compared with the original version to identify items or words that were not equivalent. After that, an expert panel including all the translators, content expert, language expert, and research methodologist was convened, and the final PRWE-Spanish (version 2.0) was developed by consensus.

### Assessment of PRWE-Spanish measurement properties

The final PRWE-Spanish was assessed for internal-consistency reliability, test-retest reliability, measurement error, and construct validity in patients with DRF.

#### Study design

The study was an observational study with a classic cohort design for test-retest reliability and a cross-sectional design for the construct validity analysis, which adhered to the STROBE guidelines [30].

#### Eligibility criteria

The inclusion criteria were patient age ≥ 17 years, extra-articular DRF, treatment with closed reduction and cast, native Spanish (Spain) speaking, and ability to understand and respond to the questionnaires. The exclusion criteria were neurological or rheumatic disorders and concomitant traumatic lesions in the upper extremity.

#### Study participants

During a 12-month period starting in January 2015, all patients who attended the emergency department for the North region of Santa Cruz de Tenerife, Canary Islands, Spain (University Hospital of La Candelaria), with acute extra-articular DRF treated with closed reduction and cast immobilization were invited to participate. Eligible patients were recruited by specialists in orthopedics or hand surgery (RGG, LRB) after clinical and radiographic examinations. Of all patients that were eligible and invited, no patient declined to participate. Each patient was given verbal and written information about the study and informed consent was obtained. All procedures performed in this study involving human participants were in accordance with the ethical standards of the institutional national research committee of the University Hospital of La Candelaria, Tenerife, Spain, and with the 1964 Helsinki declaration and its later amendments or comparable ethical standards. Demographic data and injury related information were collected at the time of enrolment.

#### Outcome measures

The patients completed the Spanish versions of the PRWE (PRWE-Spanish), 11-item QuickDASH [29, 31] and EQ-5D [32] questionnaires at baseline (questionnaires were mailed to patients from the trauma center within 1 week of their fractures and inquired about status the week before fracture), and at 8, 9, 12, and 13 weeks (the questionnaires were completed by the patients at the outpatient clinic). It was not necessary to provide assistance to the respondents because the questionnaires were suitable for self-administration and the instructions were clearly written at the beginning of every questionnaire.

The PRWE consists of 2 subscales: pain (5 items) and function (10 items). A pain score (PRWE-pain) is calculated as the sum of the 5 pain items and a function score (PRWE-function) is calculated as the sum of the 10 function items divided by 2. Each subscale score may range from 0 (best) to 50 (worst). The total PRWE score (PRWE total score) is the sum of the pain and function scores, ranging from 0 (best) to 100 (worst). A missing item response can be replaced with the mean score of the subscale.

The QuickDASH is the shorter version (11 items) of the 30-item DASH questionnaire developed for measuring disability related to the upper extremity. The QuickDASH is scored from 0 (no disability) to 100 (worst possible disability). At least 10 of the 11 items must be completed for a score to be calculated. Each item is scored 1 to 5 and the assigned values for all completed items are summed and averaged, producing a score of 1 to 5. This value is then transformed to a score of 0 to 100 by subtracting one and multiplying by 25. This transformation is done to make the score easier to compare to other measures scaled on a 0–100 scale [29, 31].

The EQ-5D consists of 5 items; mobility (MO), self-care (SC), usual activities (UA), pain/discomfort (PD), and anxiety/depression (AD), each scored as 1 (no problems), 2 (moderate problems), or 3 (extreme problems). The EQ-5D Index, calculated according to special algorithm, ranges from −0.59 (worst) to 1 (best health). The index cannot be calculated when responses are missing for one or more of the dimensions [32].

#### Data analysis

For the correlation hypotheses we considered the correlation as moderate when Spearman correlation coefficient ( *r)* > 0.40 and strong when *r* > 0.70 [33]. A priori sample size calculation for the correlation analysis showed that based on the proposed null hypothesis (Ho = the correlation is equal to zero), with 0.05 significance level, 80% power and expected minimum r of 0.4, a sample size of 37 patients would be needed. The data were analyzed for normal distribution using the Shapiro-Wilk test (level of significance of 0.05), Q-Q plot and histogram. Mean scores and SD were calculated. Internal-consistency reliability was assessed with the Cronbach alpha coefficient (alpha >0.7 considered as good internal consistency) [34, 35]. Test-retest reliability was analyzed with the ICC_2,1_ [26, 35, 36] using two-way random effect model and absolute agreement definition, between responses at 8 weeks and 9 weeks and between responses at 12 weeks and 13 weeks after fracture (ie, 1 week washout time). For ICC interpretation, values greater than 0.75 were considered as indicating excellent agreement [36].

Cross-sectional precision was analyzed with the Standard Error of the Measurement (SEM = SD multiplied by the square root of (1 – Cronbach alpha)). Longitudinal precision for the test-retest reliability coefficient was analyzed with the Standard Error of the Measurement difference (SEMdiff = SD multiplied by the square root of (1 – ICC) multiplied by square root of 2) and the Minimal Detectable Change at 90% confidence level (MDC_90_ = SEM diff multiplied by 1.65) and 95% confidence level (MDC_95_ = SEM diff multiplied by 1.96) [26, 29].

For the assessment of construct validity we hypothesized that the PRWE-Spanish would have a strong positive correlation with the QuickDASH and a moderate negative correlation with the EQ-5D Index at baseline and 8 weeks. The construct validity hypotheses were analyzed with the Spearman correlation coefficient (r). A *p*-value of <0.05 was used for statistical significance. For sample size calculation we used StatsToDo (https://www.statstodo.com/SSizCorr_Pgm.php) and for data analyses we used IBM SPSS Statistics v. 20.0 and STATA v. 14.2.

## Results

### **Translation and adaptation** of the PRWE to Spanish

The average rating for difficulty of translation ranged from 0 to 5 in 11 of the 15 items and the average rating for equivalence of translation was high, exceeding 86 in all items (Table 1). One minor cultural adaption was done in the Spanish (Spain) version of the PRWE; because of the metric measurement system used in Spain, we modified the item “carry a 10lb object in my affected hand”, so that the weight was expressed in Kilograms (5 Kg). No other changes were necessary in the final Spanish (Spain) version.

**Table 1:** Difficulty and equivalence of item translation of the Spanish Patient-Rated Wrist Evaluation (PRWE) as rated by four translators

Spanish PRWE	Difficulty of translation^a^	Equivalence of translation^b^
	Mean (range)	Mean (range)
Pain		
Item 1	7.5 (5–10)	94.0 (90–96)
Item 2	3.8 (0–5)	98.4 (95–100)
Item 3	5.0 (0–10)	100 (100–100)
Item 4	2.5 (0–5)	91.4 (90–95)
Item 5	0.0 (0.0)	97.0 (95–100)
Function - Specific activities		
Item 6	6.3 (5–10)	100 (100–100)
Item 7	13.8 (5–30)	86.3 (85–90)
Item 8	5.0 (0–20)	96.3 (95–100)
Item 9	11.3 (5–20)	88.8 (80–95)
Item 10	2.2 (0–5)	97.5 (95–100)
Item 11	0 (0–0)	100 (100–100)
Function - Usual activities		
Item 12	0 (0–0)	95.0 (90–100)
Item 13	0 (0–0)	96.3 (95–100)
Item 14	3.8 (0–5)	97.5 (90–100)
Item 15	1.3 (0–5)	98.9 (95–100)

^a^Scale range 0 (no difficulty) to 100 (severe difficulty)

^b^Scale range 0 (no equivalence) to 100 (complete equivalence)

### Assessment of the PRWE-Spanish

#### Patient characteristics

Forty patients (31 women) participated in the study (Table 2). Mean age of the patients was 58 years (range 17 to 90 years). The fracture involved the right radius in 23 patients (57.5%). All participants completed all follow-up evaluations.

**Table 2:** Demographic characteristics of the patients

Number of patients	40
Age, mean (SD) years	58 (18)
Gender, men: women	9: 31
Hand dominance, right: left	35: 5
Injured side, right: left	23: 17
Injured limb, dominant: non-dominant	18: 22

*SD* standard deviation

Values are numbers (n) unless otherwise specified

#### Outcomes scores

No missing item responses were observed in any measure. Table 3 shows the PRWE and QuickDASH scores and EQ-5D Index at all the assessment points. The scores of the PRWE and QuickDASH presented a similar evolution (Table 3). The mean disability scores were lowest (best) at baseline (PRWE, 9.3; QuickDASH, 11.7), peaking at 8 weeks (58.7 and 59.1, respectively), and then decreasing at 13 weeks after the injury (26.9 and 25.4, respectively). The mean EQ-5D Index was highest (best) at baseline (0.84), lowest at 8 weeks after the injury (0.55), and increased at 13 weeks (0.77).

**Table 3:** Scores for the Spanish Patient-Rated Wrist Evaluation, QuickDASH and EQ-5D

Scale^a^	Baseline	8 weeks	9 weeks	12 weeks	13 weeks
	Mean	Mean	Mean	Mean	Mean
	(SD)	(SD)	(SD)	(SD)	(SD)
PRWE					
Pain	5.0 (10.8)	28.9 (10.3)	25.6 (10.3)	15.7 (10.4)	13.9 (9.8)
Function	4.2 (10.8)	29.8 (11.2)	25.9 (11.3)	15.4 (11.9)	13.0 (11.0)
Total	9.3 (21.1)	58.7 (20.6)	51.4 (20.8)	31.0 (20.8)	26.9 (19.9)
QuickDASH	11.7 (20.2)	59.1 (20.6)	51.3 (20.3)	30.4 (21.6)	25.4 (20.4)
EQ-5D Index	0.84 (0.20)	0.55 (0.30)	0.61 (0.28)	0.75 (0.22)	0.77 (0.22)

Abbreviations: EQ-5D, EuroQoL 5 dimensions; PRWE, Patient-Rated Wrist Evaluation; QuickDASH, 11-item Disabilities of the Arm, Shoulder and Hand; SD, standard deviation

^a^Score range: PRWE pain and function subscales, 0 (best) to 50 (worst); PRWE total score, 0 (best) to 100 (worst); QuickDASH, 0 (best) to 100 (worst); EQ-5D Index, −0.59 (worst) to 1 (best)

#### Reliability and Measurement Error of the PPRWE-Spanish

At baseline, the Cronbach alpha was 0.96 (SEM = 2.13) for the pain, 0.98 (SEM = 1.56) for the function and 0.98 (SEM = 2.67) for the total scores, and the corresponding values at 8 weeks were 0.89 (3.50), 0.95 (2.58) and 0.96 (4.37), respectively (Table 4).

**Table 4:** Cross-sectional precision and Standard Error of the Measurement at baseline and 8 weeks

	Cronbach alpha	SD	SEM	Cross-sectional precision interval (95% CI)
Baseline				
Pain	0.96	10.8	2.13	+/− 4.18
Function	0.98	10.76	1.56	+/− 3.03
Total	0.98	21.09	2.67	+/− 5.23
8 weeks				
Pain	0.89	10.74	3.50	+/− 6.85
Function	0.95	11.20	2.58	+/− 5.05
Total	0.96	20.59	4.37	+/− 8.56

Abbreviations: CI, confidence interval; SD, standard deviation; SEM, Standard Error of the Measurement

Test-retest reliability showed an ICC_2,1_ of 0.93 for the pain, 0.94 for the function, and 0.94 for the total scores at the interval 8-week and 9-weeks, and 0.98, 0.90, and 0.96, respectively, for the 12-week and 13-week responses. For the interval 8 weeks and 9 weeks the SEMdiff was 4.1 (MDC_95_ = 8.04) for the pain, 3.98 (MDC_95_ = 7.79) for the function, and 7.61 (MDC_95_ = 13.74) for the total scores, and for the interval 12 weeks and 13 weeks, the SEMdiff (MDC_95_) values were 2.13 (4.17), 5.34 (10.46), and 6.18 (12.11), respectively (Table 5).

**Table 5:** Intraclass correlation coefficient, Standard Error of the Measurement difference and Minimal Detectable Change at 95% confidence level

PRWE subscale	Mean score difference (95% CI)	ICC (95% CI)	SEM_diff_	MDC (90)	MDC (95)
8–9 w					
Pain	3.28 (1.53–5.02)	0.93 (0.86–0.96)	4.10	6.77	8.04
Function	3.94 (2.18–5.69)	0.94 (0.88–0.97)	3.98	6.56	7.79
Total	7.22 (4.12–10.31)	0.94 (0.89–0.97)	7.61	11.57	13.74
12–13 w					
Pain	1.75 (0.82–2.68)	0.98 (0.92–0.99)	2.13	3.51	4.17
Function	2.42 (0.2–4.62)	0.90 (0.81–0.95)	5.34	8.81	10.46
Total	4.16 (1.48–6.84)	0.96 (0.92–0.98)	6.18	10.19	12.11

Abbreviations: CI, confidence interval; ICC, intraclass correlation coefficient; MDC (90), minimal detectable change at 90% confidence interval; MDC (95), minimal detectable change at 95% confidence level; SEM_diff_, Standard Error of the Measurement difference

#### Construct validity of the PRWE-Spanish

There was a strong positive correlation between the scores for the PRWE-Spanish and the QuickDASH at baseline (*r* = 0.71, *p* < 0.001) and at 8 weeks (*r* = 0.79, *p* < 0.001), and a moderate negative correlation with the EQ-5D Index at the baseline (*r* = −0.45, *p* = 0.004), and at 8 weeks (*r* = −0.40, *p* = 0.01) (Table 6).

**Table 6:** Construct validity assessment of the Spanish PRWE

Time	r (95% CI)^a^	t^b^	*P* value
Baseline			
QuickDASH	0.71 (0.51 to 0.84)	6.19	<0.001
EQ-5D	−0.45 (−0.16 to −0.67)	3.10	0.004
8 weeks			
QuickDASH	0.79 (0.64 to 0.88)	8.05	<0.001
EQ-5D	−0.40 (−0.11 to −0.64)	2.75	0.01

Abbreviations: CI, confidence interval; r, Spearman’s rho coefficient of correlation

^a^Correlations between PRWE score and QuickDASH score and between PRWE score and EQ-5D index at baseline and at 8 weeks

^b^Contrast statistic that follows the Student-Fisher law with n-2 degrees of freedom

## Discussion

The results of this study have demonstrated that the Spanish version (Spain) of the PRWE had good internal-consistency and test-retest reliability. The correlations were concordant with the a priori formulated construct hypotheses supporting good construct validity. The results of the adaptation process showed an equivalence in translation scores (ETS) of at least 85% and the difficulty in translation scores (DTS) ranging from 0 to 30. Similar results were reported in the Spanish adaptations of the CTS questionnaire using the same adaptation-translation method (ETS range, 85 to 100; DTS range, 0 to 20), the DASH (ETS range, 98 to 100; DTS range, 5 to 45) [1], and SF-36 (ETS range, 80 to 100; DTS range, 5 to 45) [28]. Only one PRWE item had to be modified by converting pounds to kilograms, as was done in other language versions of the PRWE [19, 24, 25]. The adaptation process used to obtain the Spanish version of the PRWE generally followed the guidelines applied to many previous cross-cultural adaptations of health status and quality-of-life measures [37].

Internal-consistency analysis demonstrated a Cronbach alpha coefficient greater than 0.7 in subscale items and total items of the Spanish PRWE both at baseline (range 0.96 to 0.98) and at 8 weeks (range 0.89 to 0.95). A similarly high internal consistency has been found in previous cross cultural-adaptations of the PRWE. Cronbach alpha values ranging from 0.89 to 0.92 have been reported for the Hindi version with a sample of 50 patients with DRF with no information about type of treatment [24], and of 0.93 to 0.95 for the Korean version in 63 patients with DRF treated with open reduction and volar plate fixation [25]. High internal consistency values for the PRWE have been shown in wrist conditions other than DRF (such as scaphoid fracture, arthritis, carpal ligament injuries, wrist synovial cyst, and other conditions), with Cronbach alpha between 0.81 and 0.98 [10, 11, 18, 19]. Cronbach alpha value of 0.8 indicates good internal consistency and value of 0.9 indicates excellent internal consistency. Although a very high Cronbach alpha may indicate item redundancy it has the advantage of yielding better cross-sectional precision for scores at the individual level [29].

Test-retest reliability analysis showed an ICC higher than 0.8 for the 8-week and 9-week responses and for the 12-week and 13-week responses. The 1-week washout time used in this analysis was similar to that used in previous test-retest reliability analyses of the PRWE, which ranged from 2 to 7 days [7, 10, 19]. The level of test-retest reliability observed for the Spanish (Spain) version total score was similar to that reported for the original version (ICC = 0.90) [7] and for most of the other language versions, ranging from 0.81 to 0.96 [10, 11, 19, 24, 25]. In our study, the test-retest reliability was high even in two different stages of the follow-up after DRF, at 8–9 weeks when higher disability is expected, and at 12–13 weeks when the disability is expected to be lower. Thus, the PRWE-Spanish yielded stable scores in the same population on 2 different occasions within a 1-week washout period during which patients were unlikely to experience substantial health changes and thus assumed to be stable [38]. Consequently, the Spanish PRWE achieved excellent reliability.

The precision of the measurement estimates the error around the observed score, either at one time point (cross-sectional precision) or over time (longitudinal precision). Cross-sectional precision estimates the measurement error based on the Cronbach alpha coefficient. In this study the SEM was 2.67 at baseline and 4.36 at 8 weeks. There is no previous information about SEM of the PRWE based on Cronbach alpha, possibly due to the fact that it is more common to calculate the SEMdiff in measurement [19, 34, 38]. The SEMdiff for the PRWE total scores in our study was 7.61 and 6.18, the MDC_90_ was 11.57 and 10.19, and the MDC_95_ was 13.74 and 12.11. These values are lower than the results shown in the study by John et al. [12] who reported a MDC_95_ of 22.5 in a sample of patients who had undergone interposition arthroplasty for thumb carpometacarpal osteoarthritis approximately 6 years earlier. Our results were similar to others described in previous versions of the PRWE. Schmitt and Fabio [13] found SEMdiff of 5.22 and MDC_90_ of 12.2 in a sample of patients with upper extremity musculoskeletal disorders. John et al. [12] reported SEMdiff of 8.12 for the PRWE total scores (10.54 for pain, and 7.81 for function) in a sample of patients with thumb osteoarthritis. In a sample of patients with DRF Mehta et al. [24] reported SEMdiff of 5.4 and MDC_90_ of 12.5.

Appropriate statistics for assessing measurement error are the limits of agreement (LoA) and the smallest detectable change (SDC) or minimal detectable change (MDC), both directly related to the SEMdiff [34, 38–40]. An important issue when we compare MDC is that this absolute reliability index, called smallest real difference (SRD) [34] or smallest detectable change (SDC) [38, 40], depends on several factors including the study population, washout interval, time point during the follow-up when the test-retest analysis was done, and the variance of the data [34].

MacDermid and Tottenham [14] demonstrated convergent validity of the original PRWE by showing a strong correlation with the DASH scores (*r* = 0.72). We found a similar correlation (*r* = 0.71) between the Spanish (Spain) PRWE and the QuickDASH at the baseline measurement. A higher correlation between the DASH and the PRWE was observed in the Swedish version in patients with DRF at 7 weeks (*r* = 0.86) [17], in the Dutch version (*r* = 0.84) [16], and in the Japanese version (*r* = 0.81) [11]. We found higher correlation (*r* = 0.79) between PRWE and the QuickDASH at 8 weeks after DRF.

We have found only one study that examined the correlation between the PRWE and QuickDASH; Sandelin et al. [21], using the Finnish version of the PRWE, observed a strong correlation at 2 and 4 months after DRF. The negative moderate correlation between PRWE Spanish (Spain) and the EQ-5D Index (−0.44 and −0.40), concordant with the pre-specified hypothesis, provides an additional support of the construct validity of the PRWE (higher disability related to wrist disorder correlates with lower quality of Life). To our knowledge no previous study has used the EQ-5D Index in construct validity analysis of the PRWE. However, many authors have used other quality-of-life measures, such as SF-36, in the construct validity hypothesis testing. MacDermid et al. [6] found a negative moderate correlation between the scores for the PRWE subscales and the SF-36 bodily pain scale ranging from −0.54 to −0.73. John et al. [12] reported a negative correlation between the PRWE total score and different physical dimensions of the SF-36 (physical functioning −0.46, role physical −0.39, physical component summary −0.54).

Assessment of construct validity should include testing hypotheses that can demonstrate the proposed construct. The two most important factors when choosing the hypotheses are the health dimension or concept measured and the direction of scoring of the measures [26]. We expected a strong positive correlation between the PRWE and the QuickDASH because both measure a similar concept (disability) and are scored in the same direction (higher score indicates more disability). We hypothesized a moderate negative correlation between the PRWE and the EQ-5D Index because they measure different but related concepts (disability and health related quality of life, respectively) and patients with high wrist-related disability are expected, to some extent, to have lower quality of life. A previous study of patients with CTS used responsiveness analysis to assess construct validity by demonstrating the hypothesis that an upper-extremity specific measure, the DASH, was expected to have lower responsiveness than a disease-specific measure, the CTS symptom severity scale, and higher responsiveness than a generic instrument, the SF-36 [41]. For the PRWE-Spanish, demonstrating the construct validity hypotheses suggests that it is a valid patient-reported measure of pain and disability related to wrist injury. The PRWE-Spanish would be an important tool that can assist researchers in evaluating outcomes and clinicians to follow patients after DRF. Our study has limitations. The scales assessing translation equivalence and translation difficulty have not been validated previously, although they have been used in similar studies. The sample size is moderate but in accordance with the sample size calculation and accepted standards. We have no data about the number of potentially eligible patients that may not have been asked about participation. Another limitation is the lack of responsiveness analysis. The results presented in this study can be generalizable only to patients with non-operatively treated DRF, because the psychometric properties of an outcome measure are context-specific.

Further studies regarding responsiveness and interpretability including determining the minimal clinically important difference are needed to complete the analysis of the measurements properties of the Spanish (Spain) PRWE. Future research direction for the PRWE Spanish and the PRWE in general should include studying its measurement properties in other wrist disorders.

## Conclusions

This study has demonstrated that the Spanish (Spain) PRWE measure has good reliability and constructs validity for outcomes assessment in non-operatively treated DRF. The reliability has been established for both subscales and for the total score, which is an important feature because it suggests that the individual subscales can provide useful and reproducible data if they are used independently. The PRWE-Spanish showed construct validity for measuring outcome at 8 and 12 weeks after DRF. The PRWE-Spanish would be a useful tool for researchers and clinicians who manage patients with wrist disorders in Spain and it contributes to the knowledge about the PRWE as a patient-reported outcome measure. This Spanish version could also be helpful in other Spanish-speaking countries that do not have own version of the PRWE but the fact that the translation and cultural adaptation was conducted in Spain should be taken into consideration when using it in other Spanish-speaking parts of the world.

## Abbreviations

CTS: Carpal Tunnel Syndrome
DASH: Disability of the Arm, Shoulder and Hand
EQ-5D: Euroqol 5 dimensions
HRQOL: Health Related Quality of Life
ICC_2,1_: Intraclass Correlation Coefficient two-way random effect model and absolute agreement definition
MDC: Minimal Detectable Change
PRWE: Patient Rated Wrist Evaluation
QuickDASH: Shorter form of the DASH
SD: Standard Deviation
SDC: Smallest Detectable Change
SEM: Standard Error of the Measurement
SEMdiff: Standard Error of the Measurement Difference
SF-36: Short form 36 items Health Survey
